# The Role of Gamma-Band Activity in the Representation of Faces: Reduced Activity in the Fusiform Face Area in Congenital Prosopagnosia

**DOI:** 10.1371/journal.pone.0019550

**Published:** 2011-05-05

**Authors:** Christian Dobel, Markus Junghöfer, Thomas Gruber

**Affiliations:** 1 Institute for Biomagnetism and Biosignalanalyis, Otto Creutzfeldt Center, Westfälische Wilhelms-Universität Münster, Münster, Germany; 2 Institute for Psychology, University of Osnabrück, Osnabrück, Germany; National Institute of Mental Health, United States of America

## Abstract

**Background:**

Congenital prosopagnosia (CP) describes an impairment in face processing that is presumably present from birth. The neuronal correlates of this dysfunction are still under debate. In the current paper, we investigate high-frequent oscillatory activity in response to faces in persons with CP. Such neuronal activity is thought to reflect higher-level representations for faces.

**Methodology:**

Source localization of induced Gamma-Band Responses (iGBR) measured by magnetoencephalography (MEG) was used to establish the origin of oscillatory activity in response to famous and unknown faces which were presented in upright and inverted orientation. Persons suffering from congenital prosopagnosia (CP) were compared to matched controls.

**Principal Findings:**

Corroborating earlier research, both groups revealed amplified iGBR in response to upright compared to inverted faces predominately in a time interval between 170 and 330 ms and in a frequency range from 50–100 Hz. Oscillatory activity upon known faces was smaller in comparison to unknown faces, suggesting a “sharpening” effect reflecting more efficient processing for familiar stimuli. These effects were seen in a wide cortical network encompassing temporal and parietal areas involved in the disambiguation of homogenous stimuli such as faces, and in the retrieval of semantic information. Importantly, participants suffering from CP displayed a strongly reduced iGBR in the left fusiform area compared to control participants.

**Conclusions:**

In sum, these data stress the crucial role of oscillatory activity for face representation and demonstrate the involvement of a distributed occipito-temporo-parietal network in generating iGBR. This study also provides the first evidence that persons suffering from an agnosia actually display reduced gamma band activity. Finally, the results argue strongly against the view that oscillatory activity is a mere epiphenomenon brought fourth by rapid eye-movements (micro saccades).

## Introduction

Advances in cognitive neuroscience were often made by combining studies of neurological patients and advanced models of normal cognitive functioning [1]. In this vein, the present study is the first to examine induced Gamma-Band Responses (iGBRs) in a reasonable large sample of persons suffering from an impairment to recognize familiar faces, i.e. prosopagnosia.

IGBRs are oscillatory bursts of brain activity (∼25–100 Hz) which, during face processing, predominately occur around 150–400 ms after stimulus onset. In contrast to the phase and latency synchronized ‘evoked’ activity the term ‘induced’ indicates that these bursts are characterized by trial-by-trial phase and/or latency fluctuations. From a functional perspective it is generally assumed that iGBRs mirror the activation of ‘cortical object representations’ driven both by sensory input and top-down processes (for review see [2]). In particular, it was suggested that those neurons, which have to be integrated to activate an object representation, synchronize their activity in the gamma band frequency range. This temporal integration mechanism selectively tags the responses of neurons that code for the same stimulus, and demarcate their responses from those of neurons activated by other cognitive demands [3], [4].

Recent findings demonstrated that iGBRs also play a special role in face processing and can be regarded as “as a new face-sensitive electrophysiological measure, alongside with the well-documented N170 ERP components” [5, p. 1985]. In contrast to the N170 – but similar to the face-sensitive N250r component and its neuromagnetic correlate [6], [7] – iGBRs are higher in response to upright compared to inverted faces [8] and more pronounced to familiar compared to unknown faces [9]. It is likely that the N170 marks an automatic, initial detection of a face, while iGBRs reflect the formation of higher-level perceptual representations of a face including familiarity information [9] - note, however, that at least some aspects of individual face categorization can be observed during the time range of the N170 [10], especially if faces are personally known [11].

These observations point towards the importance of the iGBR during face processing. However, more convincing evidence for its functional role would be provided by studying participants with disturbed face perception, namely persons suffering from congenital prosopagnosia (CP). Given that their weakness is not acquired (for review see [12]), and that no or only mild structural abnormalities have been reported in these individuals [13], [14], the electrophysiological correlates of their brain activity are not contaminated by artefacts resulting from lesions prevailing in neurological cases. Investigations with evoked responses showed that the N170 (or its magnetic counterpart the M170) in CP fails to show specificity for faces compared to objects which is typically observed in control subjects [15]–[18]. Moreover, a relief of symptoms after configural training was correlated with increased face specificity of the N170 [19]. It is unknown, however, how iGBRs alter in response to familiar and unknown persons presented in upright and inverted orientations in prosopagnosia. The familiarity manipulation taps into the weakness itself [1] (cf. [13]) whereas inversion presumably hampers the main processing mode of face perception, namely configural processing [20]. Given the results from our and other groups, we expect only weak or no interactions between these factors, e.g. we demonstrated in a companion paper [21], that persons with CP display a reduced M170 in response to these manipulations localized to mainly left occipito-temporal areas. This effect was not attenuated by familiarity or inversion. The present study is intended to extend these findings and investigate how high-frequency oscillations and their cortical generators of persons with CP and controls are influenced by familiarity and orientation.

## Methods

### Ethics Statement

The study conforms with The Code of Ethics of the World Medical Association (Declaration of Helsinki). It is covered by the ethical approval of the “Kommission der Ärztekammer Westfalen-Lippe und der Medizinischen Fakultät der Westfälischen-Wilhelms Universität Münster” (5 V Pantev from 4^th^ of January 2006). Written consent was given from all participants before the study was conducted.

### Participants, Stimuli, Procedure and Magnetoencephalographic Recordings

The detailed experimental methods are described in our companion paper [21]. The seven investigated CP subjects were additionally described in previous studies [21]–[23]. In short, all CPs suffer from an inability to recognize famous persons indexed by a standardized test (Bielefelder Famous Faces Test, [24]). They also responded slower than controls, with no overlap between groups, in a delayed matching to sample task employing faces, even though they were even more accurate. Thus, there was a remarkable speed-accuracy tradeoff in CP persons which was most likely due to the employment of time consuming feature-based strategies in this group. This issue is described in detail in our earlier publication on these participants [22]. Group differences in both, the Bielefelder Famous Faces Test and the delayed matching to sample task, were highly significant and even visible on an individual subject level. In contrast to this specific impairment for faces there was no evidence for further specific or a general visual impairment. All participants performed inconspicuously in the Visual Object and Space Perception Battery [25] and several other tests unrelated to face perception [22]. In response to the stimuli employed here the group of participants with CP recognized significantly less famous faces than controls, especially if they were shown in inverted orientation [21]. An overview of their test scores on several neuropsychological tests and experiments can be found in Table 1. A complete description of the diagnostic procedure can be found in [22].

**Table 1:** Test scores and results from neuropsychological test batteries and other experiments.

	Controls	GH	MH	XG	LO	BT	XS	KA
Visual Object and Space Perception Battery								
Screening	20	18	20	19	15	18	20	20
Incomplete Letters	20	20	20	20	20	20	20	20
Silhouettes	26	27	29	22	16	16	23	29
Object Decision	18	20	18	18	18	18	20	20
Progressive Silhouettes	8	*	4	10	9	13	6	8
Dot count	10	10	10	10	10	10	10	10
Position Discrimination	20	19	20	20	20	16	19	20
Number Location	10	10	9	10	10	10	8	10
Cube Analysis	10	10	10	10	10	10	10	10
Snodgrass Picture Naming								
Naming (% correct)	100	97	100	97	100	100	100	100
Bielefelder Famous Faces Test								
% recognized faces from visual cue	73	30	31	47	3	40	46	36
Delayed Matching to Sample of faces and glasses								
Latencies (sec): glasses	1.4	1.5	2.6	2.3	1.0	1.7	1.5	1.4
Latencies (sec): faces	1.8	2.8	4.0	4.1	3.2	4.2	2.3	2.9
% correct: glasses	95	95	100	90	95	95	90	95
% correct: faces	86	95	100	90	90	85	95	95
Other aspects of face perception								
Judgment of (% correct):								
emotional expression	99	80	93	87	93	100	93	87
gender	100	100	100	100	100	100	100	100
age	100	100	100	100	100	100	100	100
gaze direction	100	100	100	100	100	100	100	100

Data from controls as well as from GH, MH, XG and XS from all tests are taken from Dobel et al., 2007.

*G.H. was tested by a different group on an earlier occasion with the progressive silhouettes and remembered the two items.

**Figure 1:**
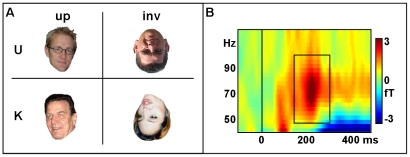
Stimulus examples and time by frequency plot. A: examples of the stimulus material (upright - up; inverted - inv; unknown -U; known –K; note that the two know persons are Gerhard Schröder and Angelina Jolie, the unknown persons are two of the authors, cd and tg). B: Grand mean baseline-corrected TF plot of the induced high frequency response averaged across all conditions, all sensors and all participants. The iGBR peak is indicated by a box (50–100 Hz, 170–330 ms).

In the current study, the seven participants with CP and seven matched controls were presented upright and inverted pictures of famous and unknown persons (66 stimuli each; each face was presented for 1000 ms in both orientations with balanced order for upright and inverted presentations). For an example of the stimulus material see Figure 1A.

Participants had to decide via button press if a face was familiar to them or not. MEG signals were recorded using a 275-sensor whole-head MEG-system (Omega 275, CTF, VSM MedTech Ltd.) with first-order axial SQUID gradiometers (2 cm diameter, 5 cm baseline, 2.2 cm average inter-sensor spacing). For further details see [21].

### Induced Gamma-Band Responses (iGBRs)

Spectral changes in oscillatory activity were analyzed by means of Morlet wavelets with a width of 7 cycles per wavelet. This procedure provides a time-varying magnitude of the signal in each frequency band, leading to a time by frequency (TF) representation of the data and is described in detail elsewhere (e.g. [2]). Importantly, TF amplitudes were averaged across single-trial frequency transformations, allowing one to analyze non phase-locked components. Furthermore, the evoked response (i.e. the ERF) was subtracted from each trial before wavelet analyses (for a similar procedure see [26], [27]).

In order to identify the latency and frequency range of the iGBR peak, mean baseline-corrected spectral amplitudes (baseline: 400 to 100 ms prior to stimulus onset) across all MEG sensors, across all experimental conditions and across all participants were represented in a TF-plot for the 40–110 Hz range.

After defining the iGBR peak (50–100 Hz, 170–330 ms), the generators of the iGBR effects were estimated by means of VARETA (Variable Resolution Electromagnetic Tomography, [28]) which is additionally described in Gruber and co-workers [29], [30] as well as Supp and colleagues [31] (2007). In brief, VARETA is based on a distributed source model with test generators at multiple cortical sites (here at 3244 voxels) serving as potential generators of the recorded signal. VARETA provides the spatial intracranial distribution of primary current densities (PCD) in source space, which is best compatible with the amplitude distribution in electrode space. Specifically, MEG epochs were transformed into the frequency domain as described above (wavelet analysis) and VARETA was applied to the complex wavelet coefficients. Due to the linear relationship between MEG and PCD, the complex source reconstructions can be interpreted as an estimate of the wavelet coefficients of the PCD (complex inverse solution; see [32]). Thereby VARETA results in localization errors of about 1–2 cm [32]. Furthermore, VARETA places anatomical constraints upon the allowable solutions. Specifically, voxels were only included for regions, in which the probability of grey matter is unequal zero (based on the Probabilistic MRI Atlas available from the MNI; [33]). Importantly, single-trial source estimates were determined for the iGBR peak time-by-frequency window and subsequently averaged across epochs.

In order to estimate significant differences between conditions, one-way ANOVA models were used. The following contrasts were analyzed: (1) upright versus inverted faces, (2) known versus unknown faces, and (3) controls versus participants with prosopagnosia. To account for spatial dependencies between voxels, activation threshold corrections were calculated by means of Random Field Theory [34]. The results were thresholded at a significance level of P<0.01. Finally, the outcomes were depicted as statistical parametric maps (SPMs) constructed on the basis of the average Montreal Brain [33].

To visualize the time-course of the iGBR signal at its cortical generators we have used a procedure suggested by Gruber and coauthors, [29]. This procedure can be summarized as follows: First the estimated brain activity (source space) for each of above contrasts (1), (2) and (3) is transferred back to MEG-sensor-space. Importantly, these forward calculations were based on only those voxels which did reveal significant effects in source space. Second, the sensors showing the greatest amplitude differences within each contrast were chosen for an optimal visualization of the time course of effects. By means of this approach we were able to identify the three sensors which were most sensitive to the three source configurations under observation. Alternatively, it would be possible to analyze the time course directly in source space. However, by projecting the signal back to sensor space, one avoids the necessity to deal with the fact that each voxel is characterized by three directions (x,y,z).

To avoid the suspicion of “double dipping”, i.e. the restriction of statistical analyses to a subset of sensors that show expected responses to manipulations [35], we do not present any statistical results of this procedure.

## Results

**Figure 2:**
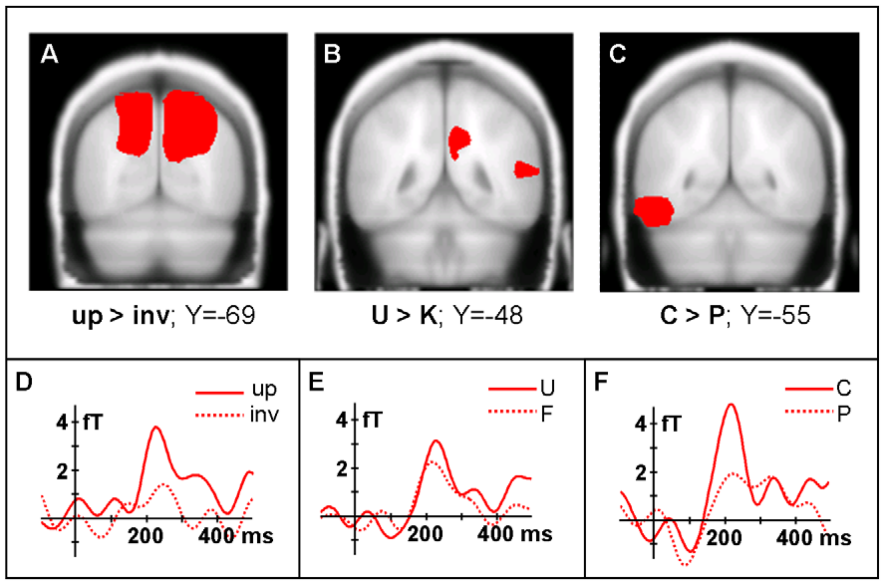
Statistical parametric maps. A, B, & C: SPMs of the inverse solutions of the iGBR peak effects. Voxels showing a significant difference are marked in red (P<0.01). Y-coordinates represent the location of the coronal slice in MNI space containing the center of gravity of the relevant contrast. A: inverted (inv) *versus* upright (up) faces. B: unknown (U) *versus* known (K). C: controls (C) *versus* participants with prosopagnosia (P). D, E, & F: Time course of the iGBR for illustrative purposes at the sensors which are most sensitive to the inverse solutions presented in A, B, and C (see text for details).

Figure 1B depicts the baseline-corrected TF-plot for the induced high-frequency range (40–110 Hz) averaged across all conditions, all sensors and all subjects. Based on this plot we have defined the iGBR peak from 50–100 Hz and 170–330 ms after stimulus onset (see box in Figure 1B). Figure 2A, B & C show SPMs for all relevant contrasts of the iGBR peak at coronal slices (the depicted slices were selected based on the centres of gravity of the contrast under observation, i.e. the slices which contain the voxel with the greatest difference between conditions).

Upright faces elicited significantly higher iGBRs as opposed to inverted faces predominantly in the left and right superior parietal lobes and occipital gyri (centre of gravity in MNI-coordinates: X: 21, Y: −69, Z: 34). As revealed by a forward solution restricted to the significant voxels depicted in Figure 1A, the left occipital sensor 125 (theta, phi [deg]: 136.2, −16.4) was most sensitive to this effect in sensor space. Unknown faces induced significantly greater Gamma-Band oscillations in the right superior parietal lobe and the right middle temporal gyrus (x,y,z [mm] centre of gravity: 14, −48, 34) as compared to known faces with the left temporal channel 123 as most sensitive sensor (theta, phi: 113.2, −15.3). IGBRs related to controls were significantly augmented in the left lateral occipitotemporal gyrus and the left inferior temporal gyrus (x,y,z: −36, −55, −10) as opposed to CPs (most sensitive left temporal sensor 120; theta, phi: 76.7, −14.5). Note that in the original study of Kanwisher and colleagues [36; p. 4306] the centre of gravity of the left fusiform face area across subjects was located in Talairach coordinates at x = −35, y = −63, z = −10.

The iGBR time course at the sensors which were most sensitive to these three source configurations is depicted in Figures 2D–F.

## Discussion

We set out to investigate iGBRs in response to faces varying in familiarity (famous/known *versus* unknown) and orientation (upright *versus* inverted) in participants with CP and unimpaired controls. We tailored the iGBR analyses to these three factors and will discuss them in turn.

First, we were able to corroborate earlier findings demonstrating higher iGBR activity in response to upright compared to inverted stimuli within the expected time interval between 150 and 400 ms (here 170–350 ms) [8], [9], [37].

This finding stands in stark contrast to the evoked activity during the M170 interval where higher activity in response to inverted faces is the typical finding (e.g. for the evoked activity of the current data [21], [11], [38], [7]). Thus, this result supports the crucial role of induced Gamma-Band activity which indexes the “activation of richer, stronger and, therefore, more easily accessible mental representations of human faces” [5, p. 1980]. The data and this interpretation is in line with the ‘representational hypothesis’ formulated by Tallon-Baudry and Bertrand, [2]. Based on the feature-binding hypothesis [e.g. 4], the “representational-hypothesis” claims that “fast oscillatory synchronization of brain areas underlies the construction of a task-relevant object representation” [2, p. 160].

The increased oscillatory activity was located in the occipital gyri, responsible for early visual processes, and, more interestingly, also in the parietal lobes. In feature conjunction tasks, areas of the superior parietal cortex have been repeatedly linked to feature integration, in the sense that lower-level features have to be spatially integrated to form a visual object [39], [31]. The parietal cortex was especially involved in feature binding when spatial information could be used to resolve ambiguity [40]. Thus, this disambiguation mechanism might be at play when visually homogeneous stimuli such as faces have to be distinguished from each other. The higher oscillatory activity upon upright faces can not be explained by more attention devoted to faces in this orientation, because attention is equally drawn to both orientations or under certain conditions even more to inverted faces [41].

Secondly, we found higher iGBR activity in the right superior parietal lobe and the right middle temporal gyrus in response to unfamiliar compared to famous faces.

Temporal lobe activity is most likely related to processing of semantic knowledge retrieval of person related information [42]. Similarly as above, we argue that the parietal activity stems from the need to resolve ambiguity especially in presence of multiple unfamiliar items.

The direction of the effect is, however, still a matter of debate. Anaki and co-authors [9] found *increased* iGBRs over frontal electrodes in response to familiar faces. Similarly, higher temporal activity was found in a functional magnetic resonance imaging (fMRI) study in response to familiar faces compared to less familiar faces [43]. In contrast, our results with *decreased* activity for familiar faces confirm a large number of studies which found reduced iGBR in response to familiar items and which proposed a “sharpening” mechanism for repeated, familiar objects [44]–[46]. This hypothesis is based on the assumption that neural assemblies coding specific stimulus features become sparser and more selective with repeated experience [47]–[51]. Reduced iGBR activity in response to repeated familiar stimuli was also found for verbal material in a study which combined electroencephalography and fMRI [26]. The “sharpening” effect is consequently not restricted to object or word recognition, but also appeared in an fMRI study investigating experience related facilitation in object naming, [52]. Thus, it constitutes a mechanism operating under various task and stimulus conditions which is observable using different methods.

Taken together it might appear as a contradiction that unfamiliar faces evoke higher iGBRs as opposed to familiar faces. However, in our understanding this demonstrates that high frequency oscillations may efficiently activate high-level object representations in two ways: either via a quantitative mechanism indexed by stronger iGBR activity or a qualitative mechanism indexed by a sharpened (or tuned) activation of neuronal populations.

This study is the first to show that persons suffering from an agnostic impairment, in our case prosopagnosia, displayed less iGBR activity. This provides further support for the crucial role of induced Gamma-Band activity for face representation and corroborates the representational hypothesis, [2]. Because we found also a smaller M170 in these subjects [21], this result is compatible with the assumption that the N170 serves to detect and categorize faces, whereas subsequent induced Gamma-Band oscillations reflect activation of their mental representations [53], [9]. In other words, we assume that the N170 is a prerequisite for subsequent Gamma-Band oscillations and we predict, at least for face processing, that Gamma-Band oscillations can not be found without an earlier N170. Given the current literature it is still an open question if the N170 and gamma band responses can be dissociated in prosopagnosic persons.

Unexpectedly, the reduced iGBR (as the M170) was seen in areas of the left hemisphere. Even though there is some evidence that individuals with CP display less left and normal right hemispheric activity [54], (for a discussion see [21]), the functional significance of that finding is not well understood. We argued in our companion paper, that the reduced left-hemispheric M170 activity is related to an overused featural processing strategy to compensate for impaired configural processing. Given the arguments above, this leads to less evoked neuronal activity, i.e. the increased usage of neuronal populations encoding featural aspects of stimuli leads to sharpened or tuned activity of such networks. As demonstrated by behavioural data and indexed by the reduced iGBRs, this compensation strategy may only be moderately successful (e.g. in situations without time pressure) and may not allow to recognize faces as effortless and automatic as it is normally the case. Nevertheless we regard it as a major challenge for researchers interested in oscillatory actitivity to investigate in future studies under which conditions and in which populations the quantitative mechanism (indexed by stronger iGBR activity) or the qualitative mechanism (indexed by a sharpened activation of neuronal populations) comes at play.

It remains an open question why we found no iGBR differences between individuals with CP and controls in the right hemisphere. Given the current literature, we see two possibilities. Either some existing accounts of the neural basis of face perception have to be challenged given impaired behaviour, but normal iGBR activity in the right hemisphere. Thus, an impairment in perceiving faces might not lead necessarily to reduced activity in the right hemisphere (see also [54]). On the other hand, our results stress the interplay of both hemispheres for successful performance in face perception. As such, the normal level of activity in CP might be related to an increased, nevertheless unsuccessful, effort of configural processing.

It has to be mentioned that the role of iGBRs in object processing has recently been challenged by the observation that gamma oscillations - measured with electroencephalography (EEG) - are not necessarily related to neural oscillations but might also arise as a consequence of miniature saccades [55]. A crucial point in the argumentation of Yuval-Greenberg and coauthors concerns the EEG reference-dependency for the localization of iGBR activity. Application of an average reference in comparison to a nose reference caused a frontal distribution of iGBR activity in the region of the eyes. In the study at hand we used the reference independent magnetoencephalography and applied a reference independent source localization method. Nevertheless, strongest induced gamma band brain activity and strongest iGBR differences across conditions and groups were observed in posterior regions while induced ocular activity in the gamma band was negligible. In addition, even though we analyzed peak activity of the iGBR, Figure 1B shows that the induced activity was rather sustained and comparable to the iGBRs reported for instance by Fries and colleagues [56, Figure 1, p. 304] and not restricted to a transient peak as in the study by Yuval-Greenberg and co-workers [55, Figure 1, p. 430]. Thus, we feel safe to say that we face a true phenomenon of oscillatory brain activity and not an epiphenomenon brought about by eye movements.

In sum, our experiment underpins the crucial functional role of iGBRs for face representations by adding evidence from persons with an agnosic impairment. These oscillations seem to serve as a fundamental computational mechanism for the selection and integration of distributed neural activity. Further studies on individuals with different perceptual impairments should make use of the powerful method to combine clinical cases with up-to-date neuroscientific methods.
